# Percutaneous Transmesenteric Access for the Embolization of Bleeding Jejunal Varices at the Hepaticojejunostomy Site: A Case Report

**DOI:** 10.7759/cureus.76731

**Published:** 2025-01-01

**Authors:** Yasuyuki Onishi, Hironori Shimizu, Ayako Iwano, Tomoaki Matsumori, Yuji Nakamoto

**Affiliations:** 1 Diagnostic Imaging and Nuclear Medicine, Kyoto University, Kyoto, JPN; 2 Gastroenterology and Hepatology, Kyoto University, Kyoto, JPN

**Keywords:** bleeding, embolization, hepaticojejunostomy, jejunal varices, transmesenteric

## Abstract

A 78-year-old man with a history of pancreaticoduodenectomy for ampullary cancer presented with bleeding jejunal varices at the hepaticojejunostomy site. Computed tomography revealed long-segment occlusion of the proximal superior mesenteric vein. Recanalization and stenting of the occluded vein were considered difficult. The varices were continuous with a jejunal vein, which was clearly visible and compressible on ultrasonography. The jejunal vein was percutaneously punctured, and the varices were embolized using 5% ethanolamine oleate via a 6-F introducer. Hemostasis was achieved using ultrasound-guided compression. No bleeding complications were observed. Thus, percutaneous transmesenteric access is a viable option for the embolization of jejunal varices at the hepaticojejunostomy site.

## Introduction

Extrahepatic portal vein stenosis or obstruction after pancreaticoduodenectomy can cause jejunal varices at the hepaticojejunostomy site [1]. Treatment for jejunal varices at the hepaticojejunostomy site includes pharmacological treatment, endoscopic management, transcatheter embolization, portal venous stenting, and surgical procedures [1]. Embolization of jejunal varices at the hepaticojejunostomy site requires careful study of the afferent and efferent veins. Access to the afferent veins is obtained through the portal venous system, either by percutaneous transhepatic, transjugular intrahepatic, or percutaneous trans-splenic route. Access is challenging when the superior mesenteric vein (SMV) is occluded. In such cases, access to one of the SMV tributaries is usually obtained after surgical exposure. Herein, we describe a case of jejunal varices embolization at the hepaticojejunostomy site via percutaneous access to a jejunal vein.

## Case presentation

**Figure 1:**
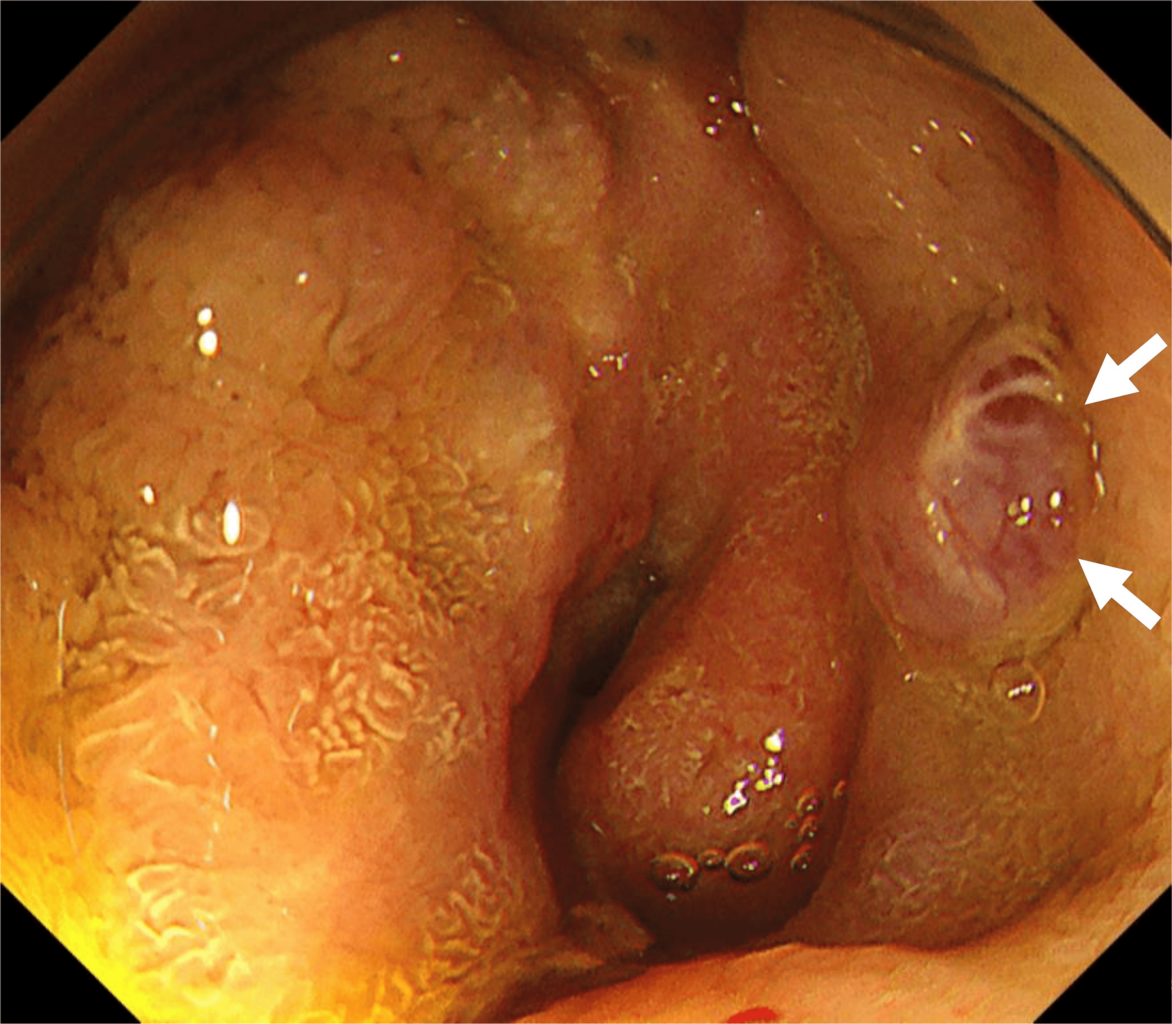
Small bowel endoscopy image using colonoscopy Varices at the hepaticojejunostomy site (arrows) are large and erythematous. They were considered unamenable via endoscopic treatment because endoscopic procedures cannot provide adequate hemostasis and may likely induce major bleeding.

A 78-year-old man underwent pylorus-preserving pancreaticoduodenectomy for ampullary cancer 27 years prior to the study. The postoperative course was complicated by pancreatic fistula and occlusion of the SMV. Herein, the patient presented with hematochezia for the first time after surgery. Laboratory tests revealed anemia (Table 1), and the patient was admitted for evaluation and treatment. Upper, lower, and capsule endoscopies did not reveal the source of the bleeding. Small-bowel endoscopy using a colonoscope (PCF-H290TI, Olympus Medical System, Tokyo, Japan) demonstrated jejunal varices at the hepaticojejunostomy site. Active bleeding was not observed. The varices were accompanied by nipple-like protrusions and erythema (Figure 1).

**Table 1:** Blood test results AST: aspartate transaminase, ALT: alanine transaminase, LDH: lactate dehydrogenase, ALP: alkaline phosphatase, Alb: albumin, CRP: C-reactive protein

Test	Result	Reference range
White blood cells	5750	3.3–8.6 × 10^3^/uL
Hemoglobin	7.6	13.7–16.8 g/dL
Platelets	256000	158–348 × 10^3^/uL
AST	20	13–30 U/L
ALT	14	10–42 U/L
LDH	137	124–222 U/L
ALP	50	38–113 U/L
Alb	3.9	4.1–5.1 g/dL
Total bilirubin	0.5	0.4–1.5 mg/dL
CRP	0.35	≤0.14 mg/dL

**Figure 2:**
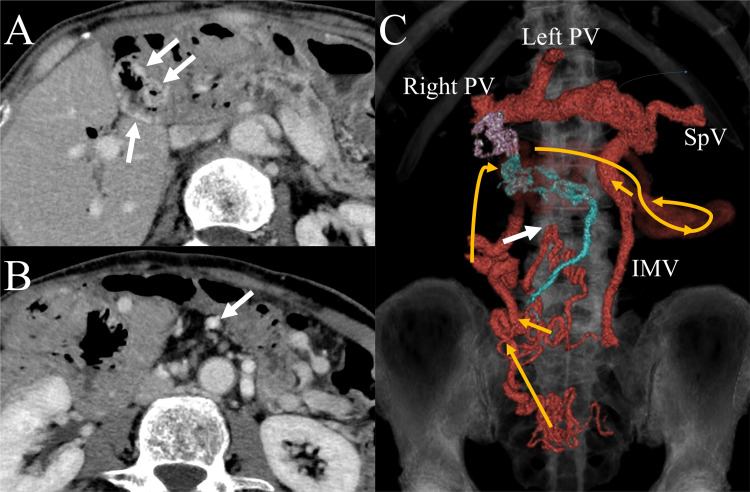
CT of the abdomen (A) Axial CT image of the upper abdomen showing jejunal varices (arrows) at the hepaticojejunostomy site. (B) Axial CT image 8 cm lower than (A) showing a continuous jejunal vein (arrow) with the jejunal varices. (C) Anterior view of three-dimensional CT showing the portal venous system. The varices (purple) are continuous with the jejunal vein (blue). The distal part of the SMV (white arrow) is visualized, but the proximal part of the SMV is occluded. Yellow arrows show the direction of the venous flow from the small intestine to the PV via the collateral vein and the inferior mesenteric vein. The central part of the collateral vein is made transparent to simplify the vascular anatomy presentation. SpV: splenic vein, CT: computed tomography, SMV: superior mesenteric vein, PV: portal vein, IMV: inferior mesenteric vein

**Figure 3:**
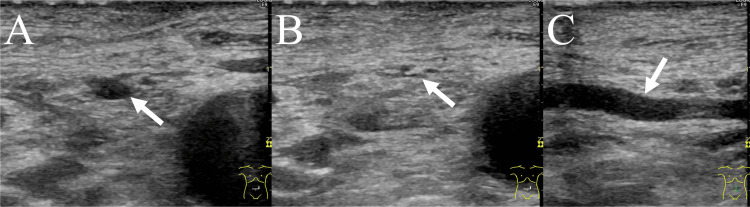
Ultrasound images of the jejunal vein continuous with the jejunal varices at the hepaticojejunostomy site (A) The ultrasound image in the transverse plane clearly visualizes the jejunal vein (arrow) without overlying bowels. (B) The jejunal vein (arrow) becomes flattened upon gentle compression. (C) Ultrasound image in the sagittal plane reveals that the jejunal vein (arrow) runs straight, and a puncture of the vein seems feasible.

Thus, jejunal-variceal bleeding at the hepaticojejunostomy site was diagnosed. Contrast-enhanced computed tomography revealed a long-distance occlusion of the proximal SMV. Most of the venous blood flow from the small bowel returned to the portal vein via a collateral vein and the inferior mesenteric vein. Jejunal varices were observed at the hepaticojejunostomy site, and a dilated jejunal vein was the afferent vein of the varices (Figure 2). On ultrasound, the jejunal vein was clearly observed without overlying bowels (Figure 3). Additionally, the vein was easily compressible because of its superficial course. As portal vein recanalization using a stent was considered difficult, we decided to percutaneously access the jejunal vein and perform transcatheter embolization of the varices.

**Figure 4:**
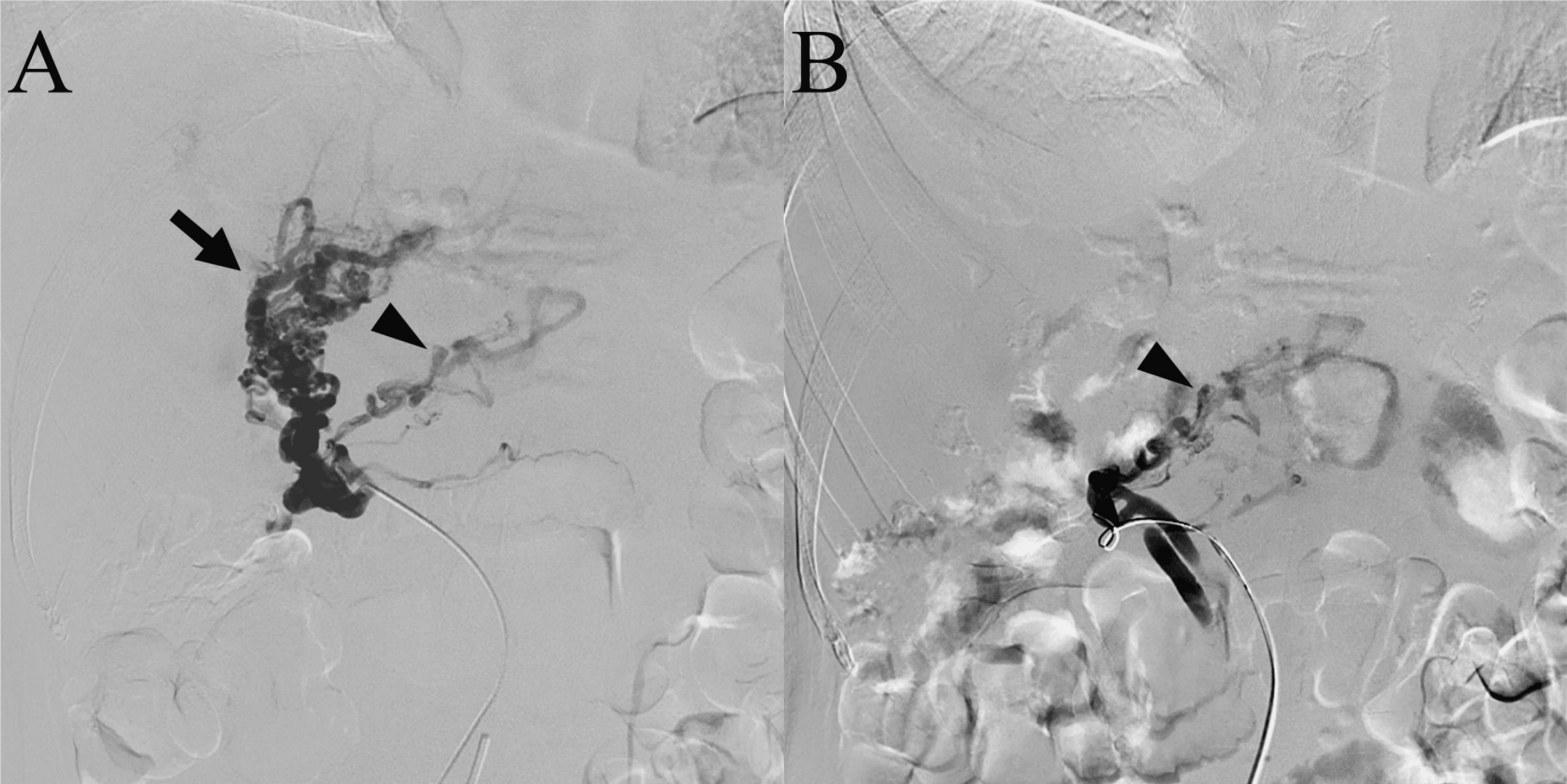
Angiography during variceal embolization via percutaneous access to the jejunal vein (A) Angiography of the jejunal vein reveals jejunal varices (arrow). A collateral vessel (arrowhead) is also observed. (B) After embolization, jejunal varices are not opacified. Note that the collateral vein (arrowhead) is preserved.

Embolization was performed under local anesthesia and moderate sedation on the 15th day after admission. The jejunal vein was percutaneously punctured using a 22-gauge Chiba needle under ultrasound guidance. A 0.018-inch wire was advanced into the jejunal vein, followed by the placement of a 6-F introducer (Merit MAK-NV, Merit Medical Systems, USA) as a 4-F sheath. Venography revealed jejunal varices and collateral veins (Figure 4A). To preserve the collateral vein, a 1.7-F microcatheter (Progreat Lambda 17, Terumo, Japan) was advanced distally into the jejunal vein branch, close to the varices. As the venous flow was slow, we decided to use ethanolamine oleate (EO) as an embolic agent without flow control, and 5% EO was slowly injected over five minutes. This injection was performed at three sites, and 6 mL of 5% EO was used. Venography revealed obliteration of the varices, with preservation of the collateral veins (Figure 4B). After sheath removal, ultrasonography-guided compression was applied to the puncture site for five minutes. Ultrasound showed no intraabdominal hematoma. The same day after the procedure, the patient developed a fever of 38.1 °C, and it subsided the next day. No complications were observed. Hematochezia did not occur, and the patient was discharged eight days after the procedure. The patient developed no bleeding episodes for three months after the procedure.

## Discussion

Several studies have highlighted the usefulness and safety of percutaneous transmesenteric access for portosystemic shunt placement in a small number of patients [2-6]. These studies involved placing a vascular sheath or dilator (4- or 5-F) or directly advancing a 4-F catheter into the mesenteric vein after obtaining transmesenteric access. Some studies did not specify the method of puncture site hemostasis [3,5,6]. In other studies, hemostasis was achieved through manual compression [2] or closure devices [4]. No bleeding complications associated with transmesenteric access occurred. In the present case, percutaneous transmesenteric access was used to embolize bleeding jejunal varices at the hepaticojejunostomy site. To date, only a few case reports have described the effectiveness of this approach for transcatheter embolization of bleeding varices [7-9]. It is worth noting that the risk of bleeding at the access site is higher when transmesenteric access is used for embolizing bleeding varices than when it is used for portosystemic shunt placement, as portal venous pressure at the puncture site decreases after successful shunt placement in the latter scenario. To reduce the risk of bleeding associated with transmesenteric access, we selected the jejunal vein, where ultrasound-guided compression is applicable. Although the safety of percutaneous transmesenteric access warrants assessment in a larger patient cohort, we believe that this approach can be applied for the embolization of bleeding varices when the target vein for puncture is compressible under ultrasound guidance.

## Conclusions

Here, we report a case of bleeding jejunal varices at the hepaticojejunostomy. Percutaneous access to the jejunal vein was performed, and the varices were successfully embolized. This case highlights the usefulness of percutaneous transmesenteric access for the embolization of jejunal varices at the hepaticojejunostomy.
